# Supplementary material for: ARG1 mRNA Level Is a Promising Prognostic Marker in Head and Neck Squamous Cell Carcinomas
Source: Diagnostics (Basel). 2021 Mar 31;11(4):628. doi: 10.3390/diagnostics11040628 (PMC8065482; doi:10.3390/diagnostics11040628)
Supplement: Supplementary file 1 [file diagnostics-11-00628-s001.zip › Supplementary Files/Supplementary files.docx]

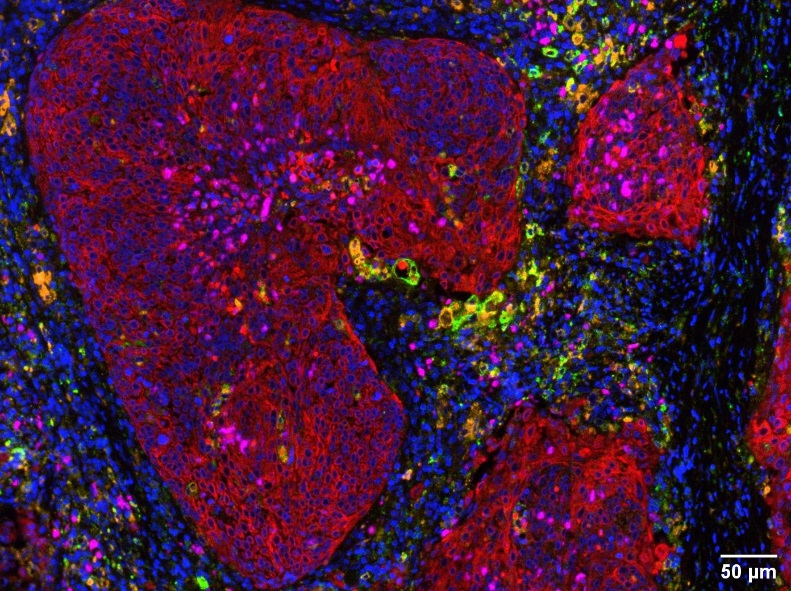

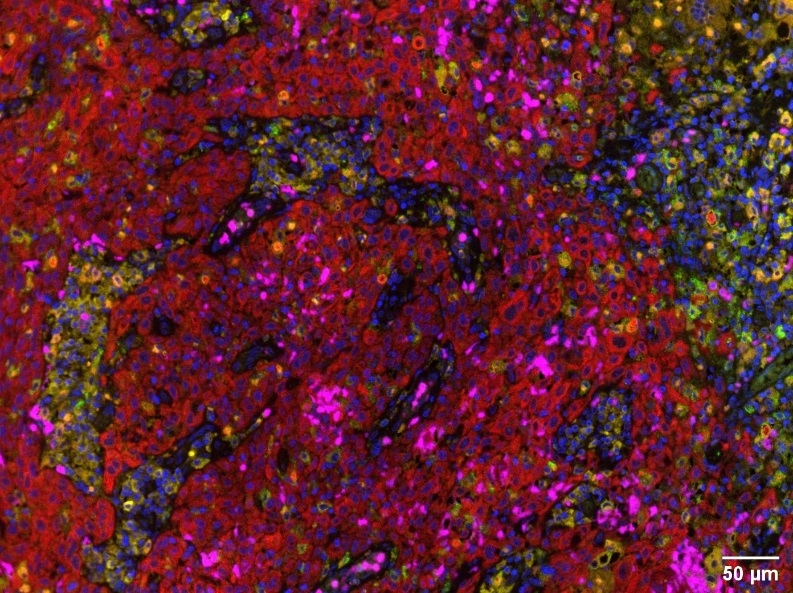


**Figure S1:** Detection of M1 and M2 macrophages in the tumor parenchyma and stroma using fIHC. Representative pictures of HPV+ (left) and HPV- (right) tumors stained with CD68 (orange), CD80 (yellow), CD163 (green), ARG1 (magenta), and Cytokeratin Pan Type I/II (red) antibodies and DAPI (blue). The parenchyma/stroma segmentation was performed according to a cytokeratin positivity/negativity, respectively. Pictures were snapped using the Mantra Snap 1.0.3. software (Akoya Biosciences, Menlo Park, CA, USA) with a magnification of 20×10 and analyzed with the InForm 2.4.6. software (Akoya Biosciences, Menlo Park, CA, USA) with prepared algorithm; the fluorophores intensities are normalized for exposure times. The scalebars represent 50 μm and were added in the Fiji (ImageJ) software.


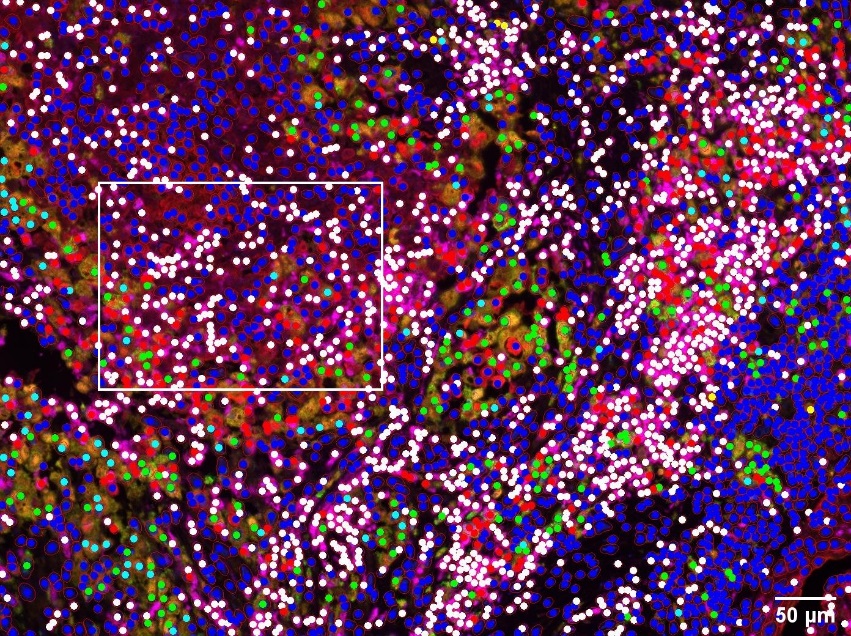
**
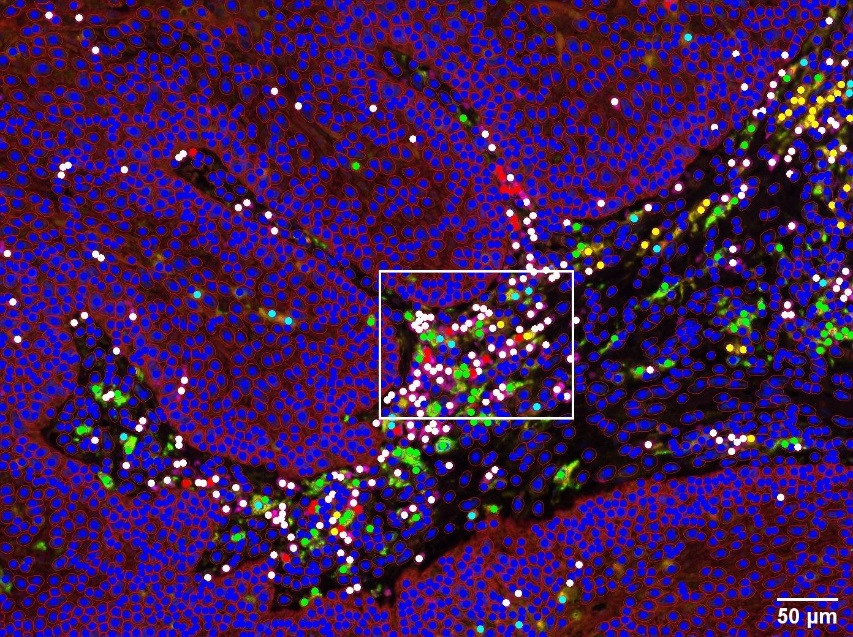
**
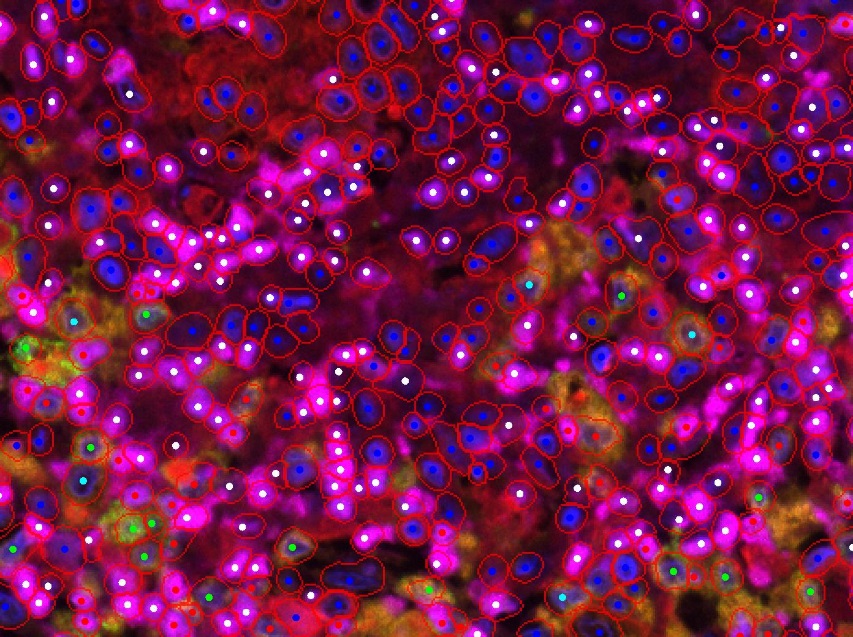

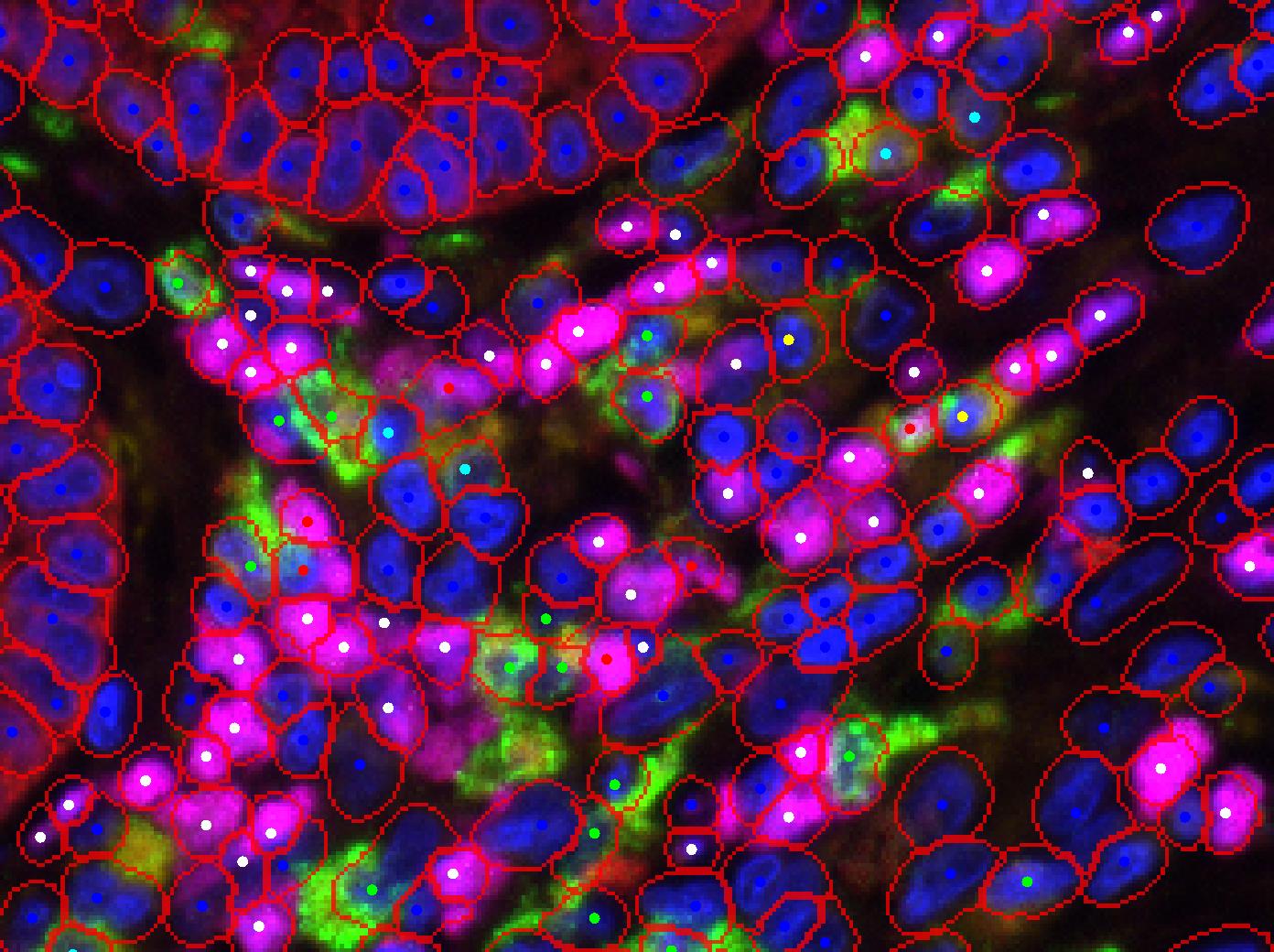


**Figure S2:** Cell phenotyping. Different cell populations were segmented and phenotyped using InForm 2.4.6. software (Akoya Biosciences, Menlo Park, CA, USA) with prepared algorithm. From original 20x10 pictures, representative cutouts were done in stroma (upper, cytokeratin negative area) and parenchyma (lower, cytokeratin positive area) in InForm 2.4.6. Red circles represent cell membrane. M1 (CD68+CD80+) – cyan dot, M2 (CD68+CD163+) – green dot, M2-ARG (CD68+ARG1+) – red dot, ARG1 (ARG1+) – white dot, CD80+ (CD80+) – yellow dot, other phenotypes – blue dot. The fluorophores intensities are normalized for exposure times. The scalebars represent 50 μm and were added in the Fiji (ImageJ) software.


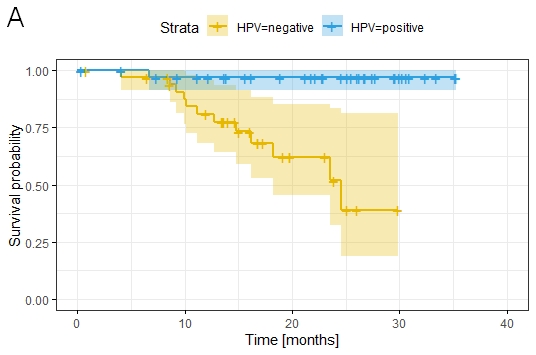


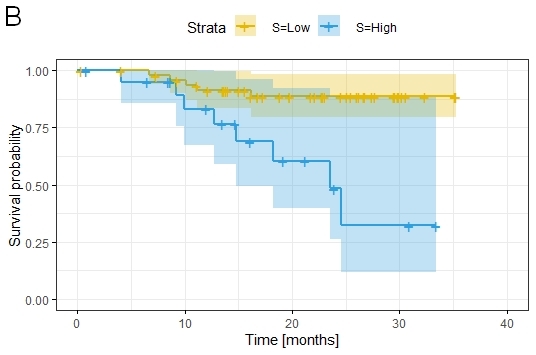


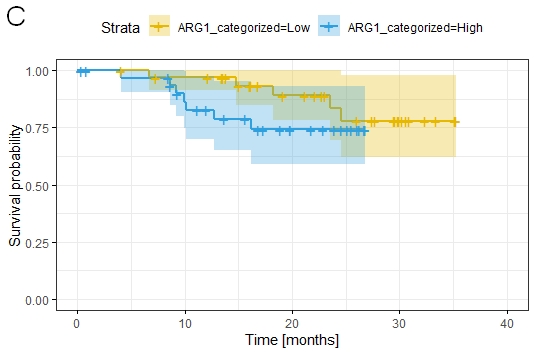


**Figure S3**: Kaplan-Meier estimator plots of A) HPV+ and HPV- patients, B) of tumor stage (S), where “low” represents S I + II, and “high” S III + IV, and C) of ARG1 mRNA level which was divided into two equal halves – “low” and “high” with threshold value 6.26.
