# Supplementary figures and images for: ARG1 mRNA Level Is a Promising Prognostic Marker in Head and Neck Squamous Cell Carcinomas
Source: Diagnostics (Basel). 2021 Mar 31;11(4):628. doi: 10.3390/diagnostics11040628 (PMC8065482; doi:10.3390/diagnostics11040628)

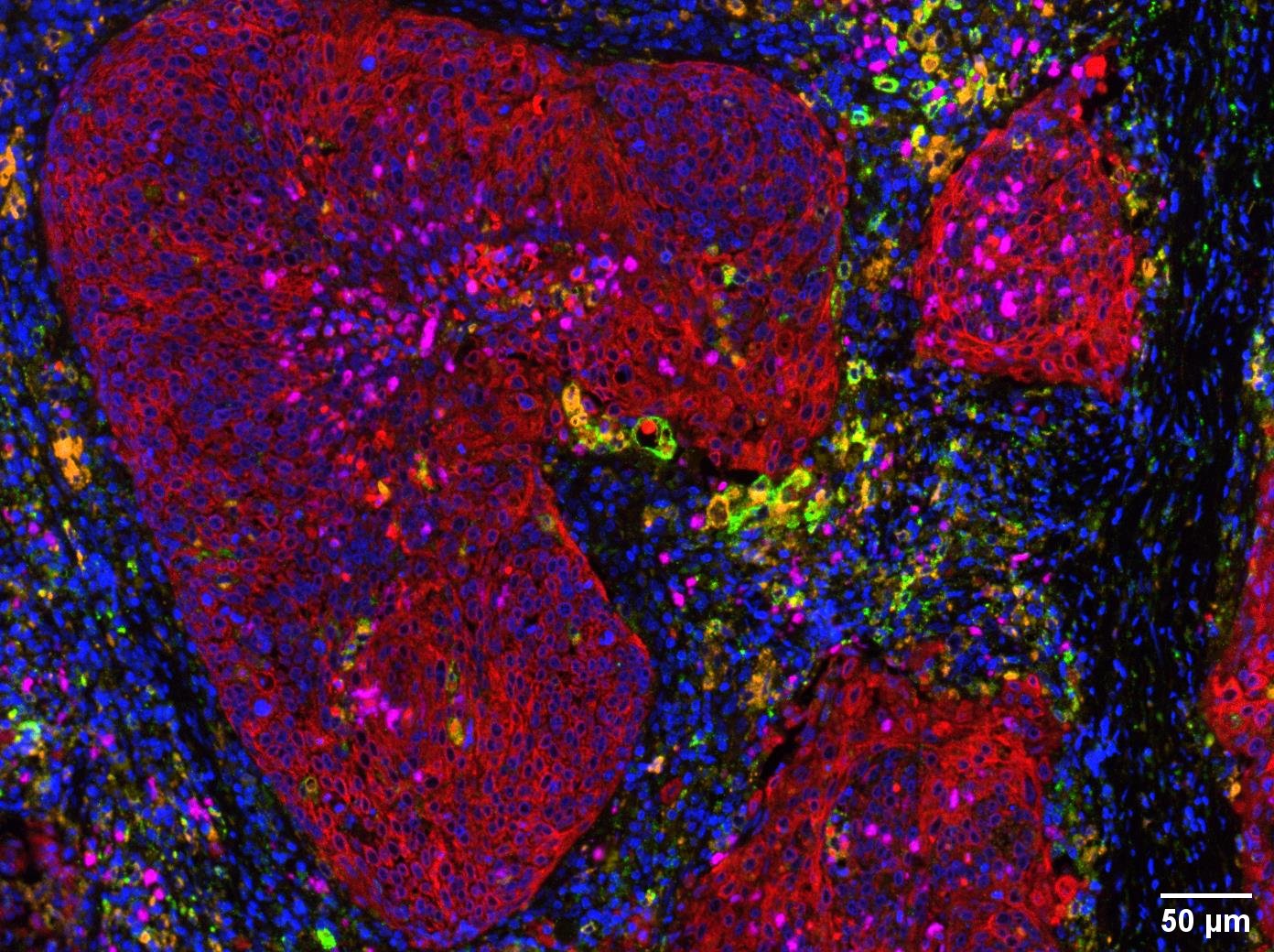

Supplement: Supplementary file 1 [file diagnostics-11-00628-s001.zip › Supplementary Files/Figure S1 left.jpg]

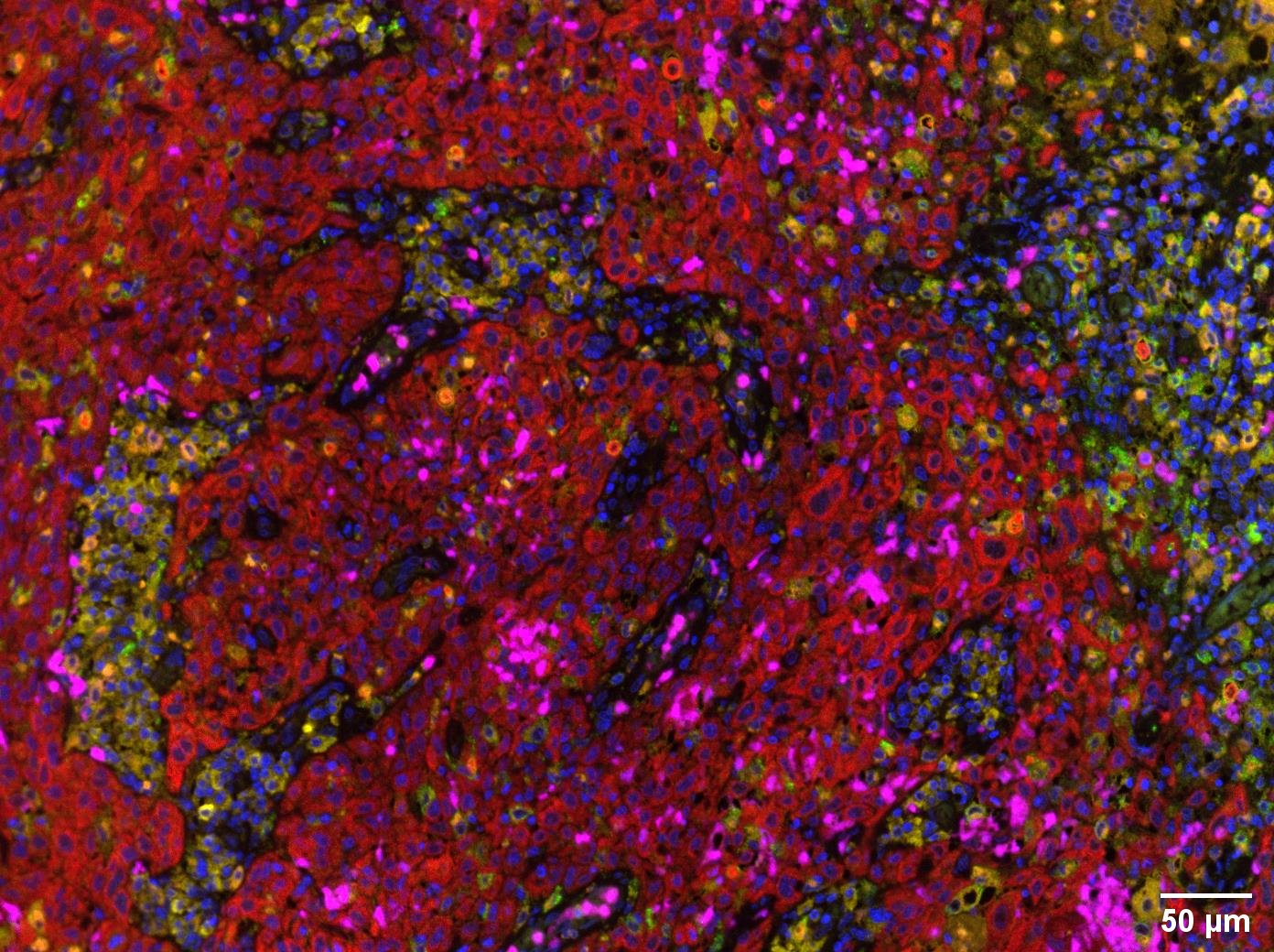

Supplement: Supplementary file 1 [file diagnostics-11-00628-s001.zip › Supplementary Files/Figure S1 right.jpg]

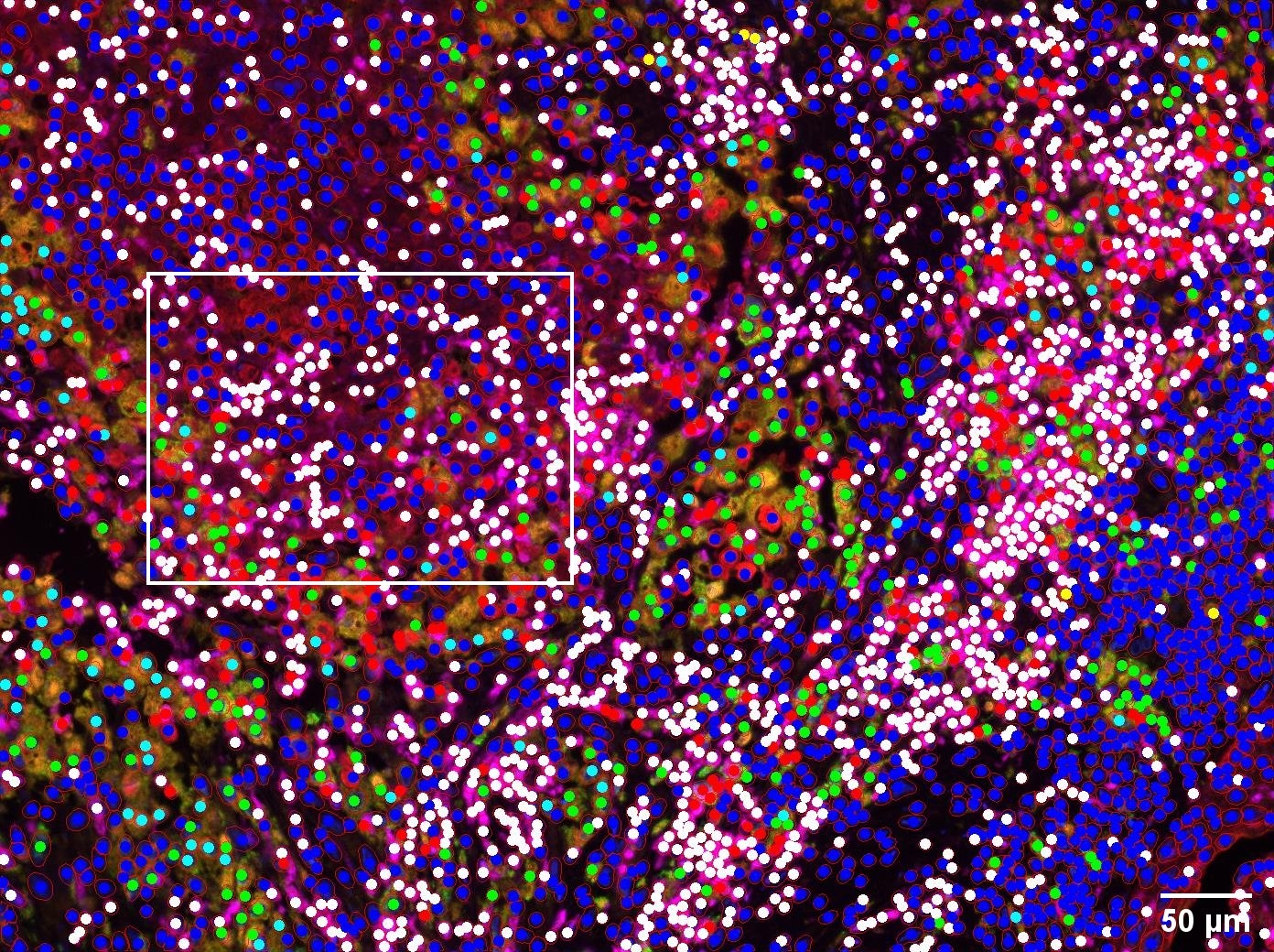

Supplement: Supplementary file 1 [file diagnostics-11-00628-s001.zip › Supplementary Files/Figure S2 lower left.jpg]

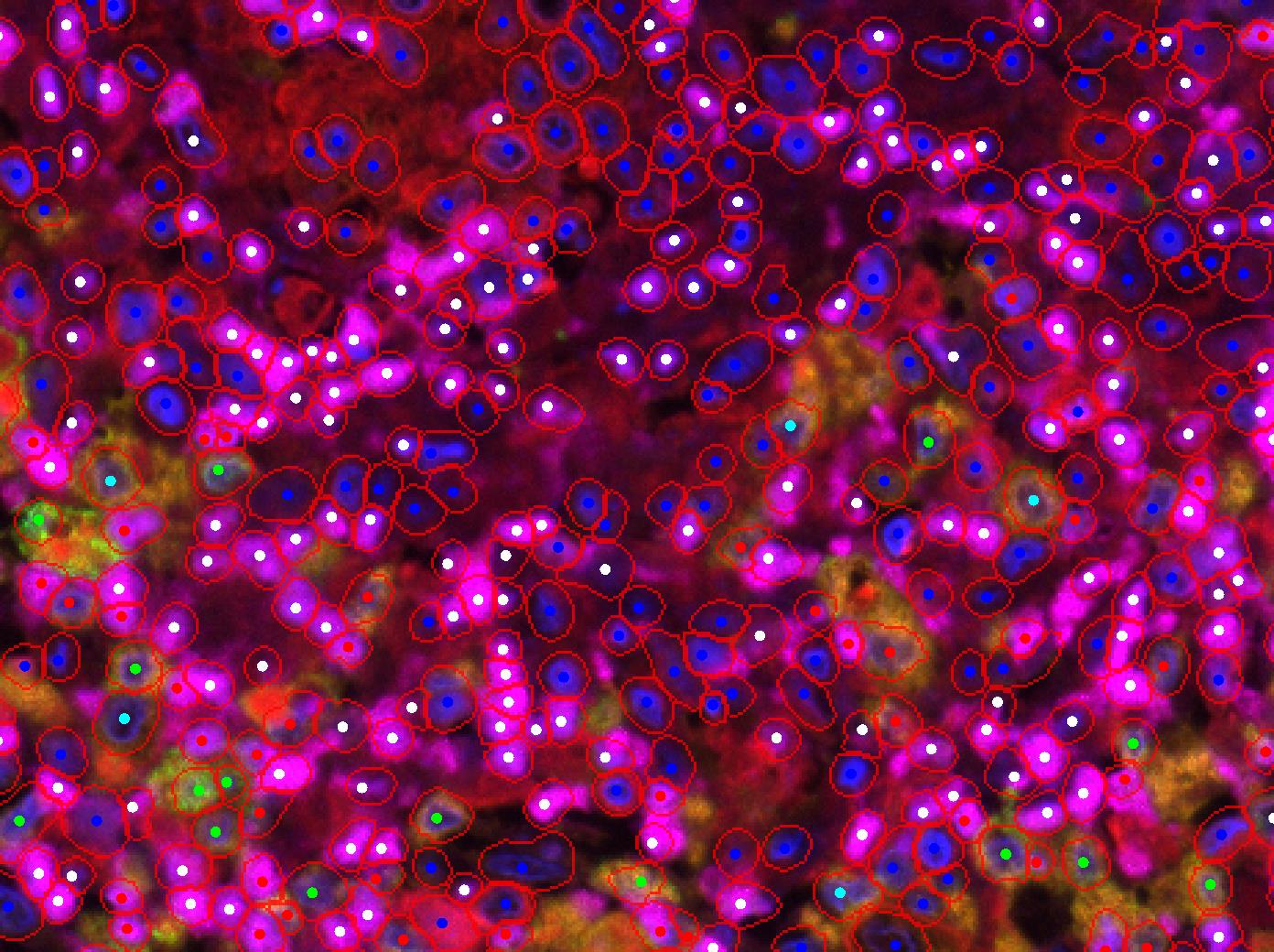

Supplement: Supplementary file 1 [file diagnostics-11-00628-s001.zip › Supplementary Files/Figure S2 lower right.jpg]

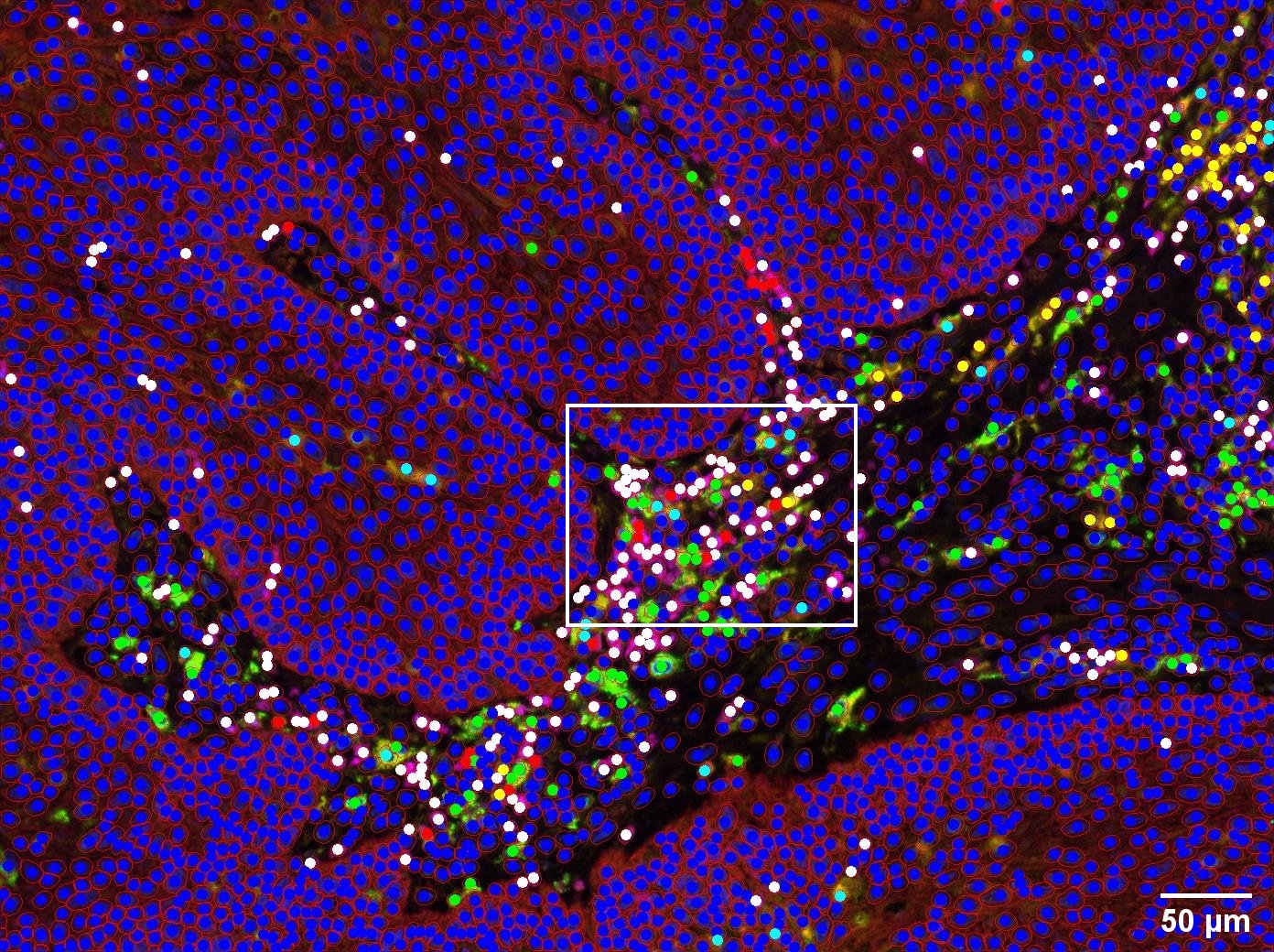

Supplement: Supplementary file 1 [file diagnostics-11-00628-s001.zip › Supplementary Files/Figure S2 upper left.jpg]

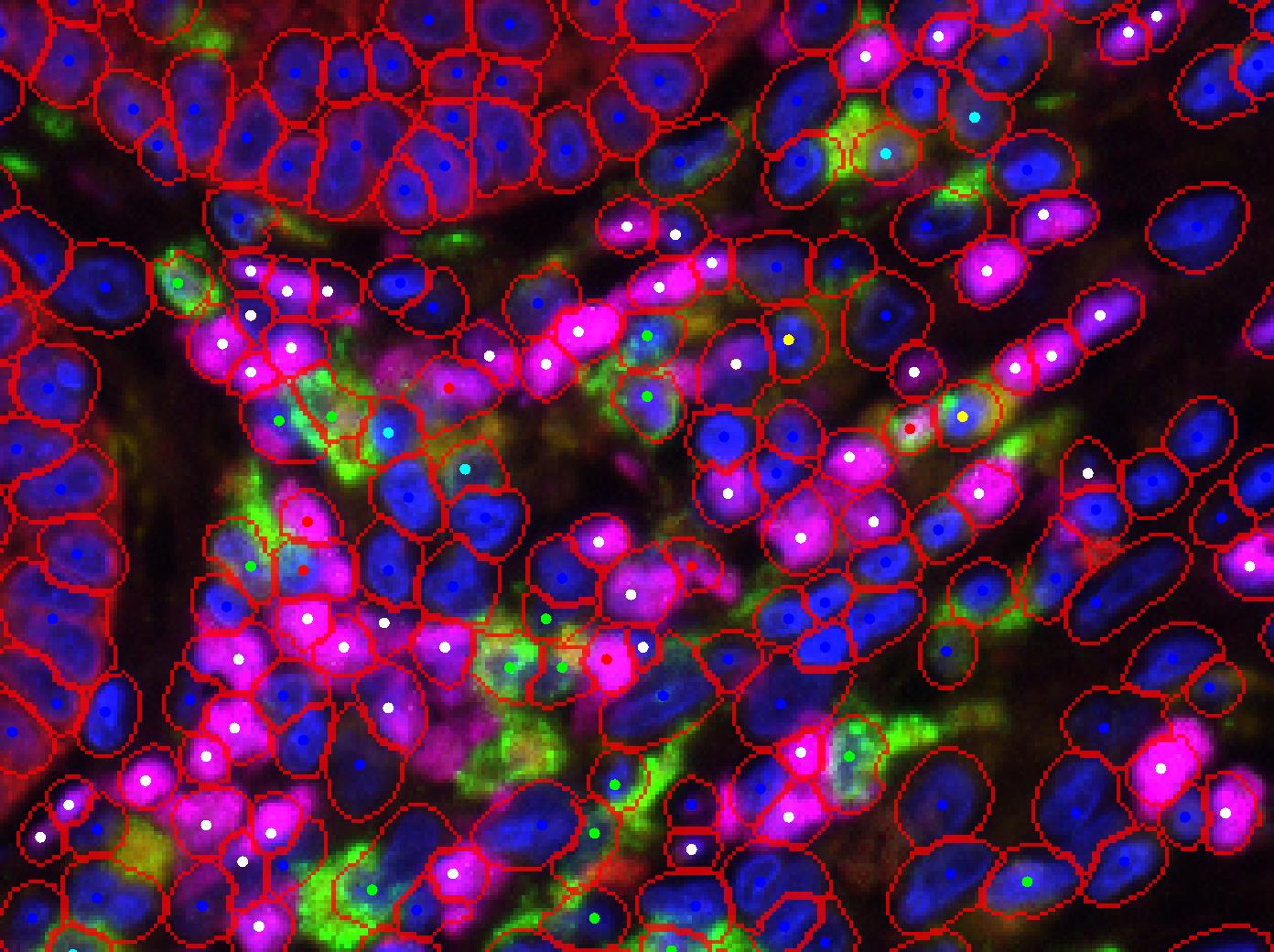

Supplement: Supplementary file 1 [file diagnostics-11-00628-s001.zip › Supplementary Files/Figure S2 upper right.jpg]

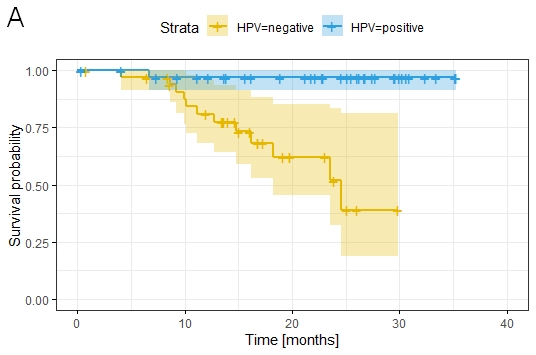

Supplement: Supplementary file 1 [file diagnostics-11-00628-s001.zip › Supplementary Files/Figure S3 A.jpg]

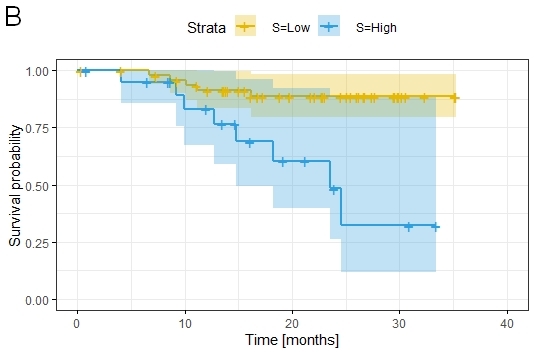

Supplement: Supplementary file 1 [file diagnostics-11-00628-s001.zip › Supplementary Files/Figure S3 B.jpg]

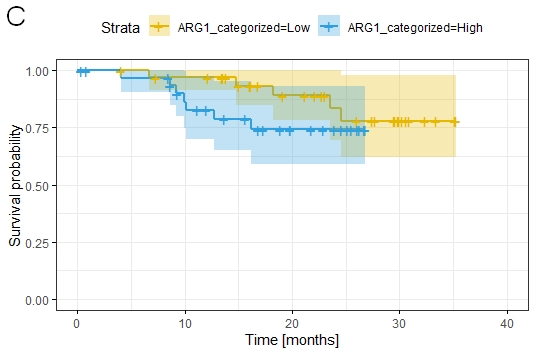

Supplement: Supplementary file 1 [file diagnostics-11-00628-s001.zip › Supplementary Files/Figure S3 C.jpg]
